# Content validation of a clinical assessment instrument for stair ascent and descent in individuals with hemiparesis

**DOI:** 10.1590/bjpt-rbf.2014.0052

**Published:** 2014

**Authors:** Mavie A. Natalio, Christina D. C. M. Faria, Luci F. Teixeira-Salmela, Stella M. Michaelsen

**Affiliations:** 1 Programa de Pós-graduação em Ciências do Movimento Humano, Universidade do Estado de Santa Catarina (UDESC), Florianópolis, SC, Brasil; 2 Departamento de Fisioterapia, Universidade Federal de Minas Gerais (UFMG), Belo Horizonte, MG, Brasil

**Keywords:** physical therapy, stroke, evaluation, biomechanics

## Abstract

**Background:**

Among the current instruments used to assess stair ambulation, none were observed that specifically evaluated the quality of movement or biomechanical strategies adopted by stroke patients.

**Objective:**

To evaluate the content validity of a clinical instrument designed to identify the qualitative and kinematic characteristics and strategies adopted by stroke patients during stair ascent and descent.

**Method:**

The first developed version, which comprised 80 items, had its content evaluated by an expert panel, which was composed of 9 well-known national and international professionals who are involved in stroke rehabilitation. The content validity index (CVI) and modified Kappa coefficients were employed for the statistical analyses. The items that demonstrated a CVI≥0.80 and Kappa≥0.75 were considered valid.

**Results:**

The content validation was performed in three stages. The final version of the instrument consisted of 38 items, which were divided into descriptive (8 items), a General Characteristics Domain (16 items) and adopted strategies (14 items) during stair ascent and descent. The total scores ranged from zero to 70 and zero to 74 for ascent and descent, respectively. Lower scores corresponded with better performance.

**Conclusion:**

Despite the satisfactory results obtained during the process of content validation, other psychometric properties of the instrument are necessary and must be evaluated.

## Introduction

The ability to ascend and descend stairs is considered a key indicator of functional independence^1^ ^-^ ^7^. Individuals with hemiparesis due to stroke report difficulty in performing this activity, even one year after the episode^8^ ^-^ ^11^. Although the kinematic gait characteristics of patients with hemiparesis have often been described in the literature^12^ ^-^ ^15^, a complete and comprehensive assessment instrument for stair ascent/descent has not been developed.

An assessment of ascending/descending stairs in a population with hemiparesis has been addressed in isolation using several instruments that separately measure items including the total ascent/descent time^3^ ^,^ ^16^, cadence^7^, handrail use^17^ ^-^ ^19^, type of step^17^ ^-^ ^19^, need for external assistance^4^ ^,^ ^17^ ^-^ ^22^, level of difficulty^4^ ^,^ ^23^ ^,^ ^24^, and use of an auxiliary device^4^, among others. However, these characteristics have not been jointly evaluated using any instrument. Although there are instruments that evaluate qualitative kinematic gait characteristics^25^ and tests such as the Timed Up and Go (TUG) test^26^ ^,^ ^27^, no available instrument evaluates the strategies used to ascend/descend stairs. Thus, through the instrument developed in the present study^28^, it was hoped that it would be possible to identify not only descriptive items (such as cadence or need for assistance) and general characteristics (such as the use of an auxiliary device or handrail) but also strategies adopted by individuals with hemiparesis who can ascend/descend stairs with/without external assistance (orthoses and/or help from another person). An activity performance analysis was conducted using video and allowed more consistent input to identify key deficits in stair ascent/descent and guided the best strategies for stroke motor rehabilitation.

The first version of the instrument consisted of 80 items described in three domains: general characteristics, functional performance, and strategies adopted in the ascent and descent of stairs. Each item was developed from an extensive literature search, expert opinion and a qualitative kinematic analysis of the ascent/descent of stairs^28^. However, to be eligible for application, all items and the entire instrument were evaluated and validated by a panel of experts^29^ ^,^ ^30^.

A content validation certifies whether an instrument measures what is intended and verifies whether the items properly reflect the content domain of interest and whether the scale dimensions are consistent with each proposed item and fulfill the specific objectives index^29^ ^,^ ^31^. Content validity aspects include the appropriateness, clarity and comprehensiveness of items, which are classified by an evaluation of the instrument items by a group of experts with previous experience or currently recognized competence in the field of study, namely judges or experts^29^ ^,^ ^32^ ^-^ ^34^. Despite its great importance, the process of content validation of original instruments is poorly described in the literature, which most often reports on the translation and cross-cultural adaptation of instruments. Therefore, the present study aimed to validate the content of a developed instrument to assess the qualitative kinematic characteristics and strategies adopted in the ascent and descent of stairs by individuals with hemiparesis.

## Method

The stages for the development and validation of an instrument encompass four distinct phases: planning, construction, quantitative analysis, and validation^29^. The instrument planning and construction phases, described by Natalio et al.^28^, consisted of an extensive literature review, expert opinion and qualitative kinematic analysis of stair ascent/descent by individuals with hemiparesis because of stroke. Content validation is described in the present study. This project was approved according to protocol no. 42/2008 of the Human Research Ethics Committee, Universidade do Estado de Santa Catarina (UDESC), Florianópolis, Santa Catarina state, Brazil.

### Participants

According to the protocol described by Polit et al.^33^, 8 Brazilian and Canadian researchers, with proven scientific track records in the field of motor rehabilitation of individuals with hemiparesis and knowledge of the biomechanics of stairs ascent and descent, were invited to participate in the present study. The length of experience of the participants ranged from 8 to 25 years, and among the 7 researchers who agreed to participate, two had Master's degrees and 5 had Doctoral degrees in the area of study. The experts reported having an excellent or good knowledge of hemiparesis, motor rehabilitation and biomechanics of stair ascent and descent. In the first phase of content validation, 7 experts, four Brazilians and three Canadians, responded within a specified period. In the second and third phases, 5 specialists participated: two Brazilians and three Canadians.

### Procedures

Three phases of content validation were performed, in which the initial and modified versions of the instrument were subjected to content validity by a committee of 7 experts with representation and recognition in the area of interest of this study. Thus, a content validation questionnaire was developed in both Portuguese and English, which evaluated the consistency, representativeness, relevance and clarity of each item developed^26^ ^,^ ^27^ ^,^ ^32^ ^-^ ^35^.

### Statistical analysis

The content validity of the developed instrument was statistically analyzed by a Content Validity Index (CVI). To calculate the CVI, each item was ranked on a four-point scale (1 = not relevant, 2 = somewhat relevant, 3 = quite relevant, 4 = highly relevant). For each item, the CVI was calculated as the number of experts who provided a rank of 3 or 4, divided by the total number of experts. The Kappa Modified Coefficient was used to determine the degree of relevance agreement of the CVI and was calculated according to Polit et al.^33^.

Seven experts participated in the first phase of content validation, the acceptable CVI value for each item ranged from 1.00 to 0.71, and the Kappa Modified value from 1.00 to 0.65. In the second and third phases, the items considered as acceptable were those that had a CVI ranging from 1.00 to 0.80 and a Modified Kappa value ranging from 1.00 to 0.76 because 5 specialists participated in these phases^31^. Because the CVI considered the number of experts consulted for each phase, the difference in the number of experts in the different content validation phases of the present study did not influence the statistical results.

## Results

The first version of the instrument was developed with a total of 80 items divided into three domains: general characteristics (6 items), functional performance (23 items), ascent strategies adopted (25 items) and descent strategies adopted (26 items). The functional performance domain presented 18 common items for the ascent and descent of stairs, in addition to two items related to the ascent and three items related to the descent of stairs. In the adopted strategies domain, 25 items were identical for ascent and descent; however, the items were evaluated separately at different phases of content validation^28^.

### The first phase of content validation

In the first phase of content validation, the domains were considered satisfactory (CVI between 0.71 and 1.00)^33^ for evaluating the ascent and descent of stairs, and the Adopted Strategies Domain, for both ascent and descent, demonstrated that major corrections were necessary.

Most items in the General Characteristics Domain obtained satisfactory scores (Table 1). All items in this domain were maintained in the second version of the instrument, but the first two items that assessed the number of stair steps and use of orthoses, respectively, were transferred to a descriptive domain with no specific score. The items that assessed the confidence level and functional graduation had their wording changed for improved understanding.

**Table 1:** The first phase of content validation of the General Characteristics Domain.

General characteristics – common items of stair ascent and descent									
Items	Consistency		Represent.**		Relevance		Clarity		Results
	CVI*	Kappa	CVI*	Kappa	IVC*	Kappa	CVI*	Kappa	
**1. Number of steps**	0.86	0.85	0.71	0.65	0.86	0.85	1.00	1.00	Domain change
**2. Use of orthoses**	0.86	0.85	0.86	0.85	1.00	1.00	1.00	1.00	Domain change
**3.1 Level of confidence**	0.71	0.65	0.86	0.85	0.86	0.85	**0.43**	**0.41**	Corrected
**3.2 Level of confidence** **with a handrail**	0.71	0.65	0.86	0.85	0.86	0.85	0.71	0.65	Corrected
**3.3 Level of confidence** **without a handrail**	0.71	0.65	0.86	0.85	0.86	0.85	0.71	0.65	Corrected
**4. Functional graduation**	**0.57**	**0.56**	0.71	0.65	0.86	0.85	0.86	0.85	Corrected

* CVI: Content validity index

** Representativeness/relevance to the field of interest.

Table 2 presents the common scores of the Functional Performance Domain for the ascent and descent of stairs. The initial two items, which assessed the cadence of the ascent and descent of stairs, were transferred to the descriptive domain (no score), and item 9, which evaluated the lower limb that initiates movement, was moved to the domain that separately evaluated ascent and descent. Items 2 and 8 were maintained without correction, and items 4, 6, 7 and 16 were excluded.

**Table 2:** The first phase of the content validation of the functional performance Domain - common items of stair ascent and descent.

Functional performance – common items of stair ascent and descent									
Items	Consistency		Represent.**		Relevance		Clarity		RESULTS
	CVI*	Kappa	CVI*	Kappa	IVC*	Kappa	CVI*	Kappa	
**A. Ascent cadence**	0.71	0.65	0.86	0.85	0.86	0.85	0.86	0.85	Domain change
**B. Descent cadence**	0.71	0.65	0.86	0.85	0.86	0.85	0.86	0.85	Domain change
**1. External assistance**	0.86	0.85	0.86	0.85	1.00	1.00	0.86	0.85	VALIDATED
**2. Handrail**	0.71	0.65	0.86	0.85	0.86	0.85	0.86	0.85	Corrected
**3. Intensity of handrail use**	0.86	0.85	0.71	0.65	0.71	0.65	**0.43**	**0.41**	Corrected
**4. Positioning of the upper limbs**	0.71	0.65	0.71	0.65	**0.57**	**0.56**	**0.57**	**0.56**	EXCLUDED
**5. Position of the paretic upper limb**	0.86	0.85	**0.57**	**0.56**	**0.57**	**0.56**	**0.57**	**0.56**	Corrected
**6. Balance reactions**	**0.57**	**0.56**	**0.57**	**0.56**	**0.57**	**0.56**	**0.43**	**0.41**	EXCLUDED
**7. Balance of the upper limbs**	0.71	0.65	**0.57**	**0.56**	**0.57**	**0.56**	**0.43**	**0.41**	EXCLUDED
**8. Pace pattern**	1.00	1.00	1.00	1.00	1.00	1.00	1.00	1.00	VALIDATED
**9. Lower limb that initiates the movement**	0.71	0.65	1.00	1.00	1.00	1.00	1.00	1.00	Domain change
**10. Supporting time**	0.86	0.85	1.00	1.00	0.86	0.85	0.86	0.85	VALIDATED
**11. Relative speed of movement**	**0.57**	**0.56**	0.71	0.65	0.86	0.85	0.86	0.85	Corrected
**12. Activity execution** **strategy**	**0.57**	**0.56**	0.71	0.65	0.86	0.85	**0.43**	**0.41**	Corrected
**13. Positioning strategy of the feet**	0.71	0.65	0.86	0.85	0.86	0.85	0.71	0.65	Corrected
**14. Foothold on the step**	**0.57**	**0.56**	0.71	0.65	0.71	0.65	**0.43**	**0.41**	Corrected
**15. Initial foot contact**	**0.43**	**0.41**	0.86	0.85	0.86	0.85	0.71	0.65	Corrected
**16. Plantar support**	**0.57**	**0.56**	**0.57**	**0.56**	**0.57**	**0.56**	**0.57**	**0.56**	EXCLUDED

* CVI: Content validity index

** Representativeness/relevance to the field of interest

In the Functional Performance Domain, items related to stair ascent, item 1, which assessed the need for assistance during the ground-stair transition, was excluded, despite having CVI and Kappa values that were near acceptable for characteristics such as relevance and clarity (CVI=0.71 and Kappa=0.65). According to the experts, this item assessed aspects not exclusively related to performance on the stairs, such as the gait on a flat surface. In the identical domain, item 2, which assessed the collision of the foot with the step, did not achieve acceptable scores for the characteristics of consistency (CVI=0.57 and Kappa=0.56) or clarity (CVI=0.28 and Kappa=0.26) and required modification.

In the Functional Performance Domain, the items related to the descent of stairs, item 1, which assessed the need for assistance during the threshold-step transition, was excluded because of low CVI and Kappa values for most characteristics analyzed. Item 2, which assessed the need for assistance during the last step-ground transition had its wording changed to better adapt it to the evaluation of stair descent because it had low CVI and Kappa values for consistency and representativeness (CVI=0.57 and Kappa=0.56). Item 3, which evaluated the safety in reaching the lower step with the foot, did not have a satisfactory score for the representativeness characteristic (CVI=0.57 and Kappa=0.56) and had its wording corrected. Furthermore, a change was performed for items 2 and 3 of this domain.

Regarding the domains developed in the first version of the instrument, according to the suggestion of one of the experts, the items of the Functional Performance Domain were included in the General Characteristics Domain. Thus, the second version of the instrument was subdivided into the General Characteristics and Adopted Strategies domains.

The items of the Adopted Strategies Domain for the ascent and descent of stairs were assessed as barely relevant by two of the 7 experts. Because this domain was similarly assessed by all experts for both the ascent and descent of stairs, and because it was a long domain, we opted to unite both sections (ascent and descent) with respect to the layout of the instrument.

The items that did not reach acceptable levels in all analyzed characteristics (i.e. consistency, representativeness, relevance and clarity) by CVI and Modified Kappa Coefficient were excluded:

- Trunk: trunk and pelvis rotation in the transition phase (0.57≤CVI≤0.71);
- Hip: adduction (0.43≤CVI≤0.57);
- Knee: internal rotation (0.43≤CVI≤0.57); external rotation (0.28≤CVI≤0.57); varus (CVI=0.43); valgus (CVI=0.43);
- Joint stability: all items were excluded for both ascent and descent (CVI=0.43).

Regarding the score of this domain, a need for score standardization for all segments evaluated was observed with only one score description maintained: (0) no deviation or the deviation was small; (1) moderate deviation; and (2) severe deviation, which greatly hindered accomplishing the task.

### Second phase of contents validation

The second version of the instrument was resubmitted to the expert committee for an evaluation of 39 items. Among these items, only four did not require a content validity assessment because they had acceptable CVI and Kappa values in the first validation phase. Thus, 35 items were reassessed in the second phase of content validation.

Table 3, which describes the second validation phase of items in the General Characteristics Domain, indicates that most items achieved satisfactory CVI and Kappa values, except items 5, 6 and 9 for the relevance characteristic and items 4, 6 and 12 for the clarity characteristic. These items were corrected and subjected to a third phase of content validation.

**Table 3:** The second phase of content validation of the general characteristics domain.

General characteristics – common items of stair ascent and descent									
Item	Consistency		Represent.**		Relevance		Clarity		Results
	CVI*	Kappa	CVI*	Kappa	IVC*	Kappa	CVI*	Kappa	
**1.1 Level of Confidence**	1.00	1.00	1.00	1.00	1.00	1.00	1.00	1.00	VALIDATED
**1.2 Level of Confidence with a handrail**	1.00	1.00	1.00	1.00	1.00	1.00	1.00	1.00	VALIDATED
**1.3 Level of Confidence without a handrail**	1.00	1.00	1.00	1.00	1.00	1.00	1.00	1.00	VALIDATED
**2. Functional graduation**	1.00	1.00	1.00	1.00	1.00	1.00	1.00	1.00	VALIDATED
**4. Use of a handrail**	0.80	0.76	0.80	0.76	0.80	0.76	**0.60**	**0.54**	Corrected
**5. Intensity of use of a handrail**	0.80	0.76	0.80	0.76	**0.60**	**0.54**			Corrected
**6. Position of the paretic upper limb**	0.80	0.76	0.80	0.76	**0.60**	**0.54**	**0.60**	**0.54**	Corrected
**9. Relative speed of lower limb movement**	1.00	1.00	0.80	0.76	**0.60**	**0.54**	0.80	0.76	Corrected
**10. Body alignment**	0.80	0.76	0.80	0.76	0.80	0.76	0.80	0.76	Corrected
**11. The feet of alignment**	0.80	0.76	1.00	1.00	0.80	0.76	0.80	0.76	Corrected
**12. Foothold**	0.80	0.76	0.80	0.76	0.80	0.76	**0.60**	**0.54**	Corrected
**13. Initial foot contact**	0.80	0.76	0.80	0.76	0.80	0.76	0.80	0.76	Corrected

* CVI: Content validity index

** Representativeness/relevance to the field of interest

Table 4 shows the results of the content validity evaluation of the characteristics analyzed for each item in the General Characteristics Domain: items related to the ascent of stairs. Two items had appropriate levels of content validity, and only item 2, which assessed the collision of the foot and step, was considered to have deficits in clarity/possibility of comprehension (wording), which required correction. Similarly, the items in the General Characteristics Domain, items related to the descent of stairs, reached adequate levels of content validity. Only item 2, which assessed the safety in reaching the lowest step with the foot during stair descent, had low CVI and Kappa values for the relevance characteristics for clinical interpretation, which could be performed based on measurement, and clarity/possibility of lack of comprehension (wording).

**Table 4:** The second phase of content validation of the independent items of the stair ascent and descent.

General characteristics – items referring to stair ascent									
Items	Consistency		Represent.**		Relevance		Clarity		Results
	CVI*	Kappa	CVI*	Kappa	IVC*	Kappa	CVI*	Kappa	
**1. Lower limb that initiates stair ascent**	1.00	1.00	1.00	1.00	0.80	0.76	1.00	1.00	VALIDATED
**2. Collision of the foot with the step**	0.80	0.76	1.00	1.00	1.00	1.00	0.60	0.54	Corrected
**General characteristics – items referring to stair descent**									
**1. Lower limb that initiates stair descent**	1.00	1.00	1.00	1.00	0.80	0.76	1.00	1.00	VALIDATED
**2. Safety during stair descent**	0.80	0.76	0.80	0.76	0.60	0.54	0.60	0.54	Corrected
**3. Need for aids**	0.80	0.76	1.00	1.00	0.80	0.76	0.80	0.76	Corrected

* CVI: Content validity index

** Representativeness/relevance to the field of interest

In the Adopted Strategies Domain, all items developed for the trunk/pelvis, hip, knee and ankle reached adequate levels of content validity for all four characteristics analyzed with CVI values ranging between 0.80 and 1.00 and a Kappa ranging between 0.76 and 1.00. The only item with a low score assessed the anteversion/retroversion of the pelvis, as shown in Table 4. This item was thus excluded from the final version of the instrument.

### Third phase of content validation

Altogether, 10 items in the General Characteristics Domain were analyzed in the third phase of content validation: items common to the ascent and descent of stairs and items related to the ascent and to the descent of stairs. Table 5 shows that all items subjected to this phase were validated after the appropriate corrections.

**Table 5:** The third phase of content validation of the general characteristics domain.

General characteristics – common items of stair ascent and descent									
Items	Consistency		Represent.**		Relevance		Clarity		Results
	CVI*	Kappa	CVI*	Kappa	IVC*	Kappa	CVI*	Kappa	
**- Use of a handrail and upper limbs**	1.00	1.00	1.00	1.00	1.00	1.00	0.80	0.76	VALIDATED
**- Time of use of a handrail**	1.00	1.00	1.00	1.00	1.00	1.00	1.00	1.00	VALIDATED
**- Position of the upper limbs**	0.80	0.76	0.80	0.76	0.80	0.76	0.80	0.76	VALIDATED
**- Symmetry of the lower limb support time**	0.80	0.76	0.80	0.76	1.00	1.00	0.80	0.76	VALIDATED
**- Body alignment**	0.80	0.76	1.00	1.00	1.00	1.00	0.80	0.76	VALIDATED
**- Feet alignment on the step**	1.00	1.00	1.00	1.00	1.00	1.00	1.00	1.00	VALIDATED
**- Foothold on the step**	1.00	1.00	1.00	1.00	1.00	1.00	0.80	0.76	VALIDATED
**General characteristics – items referring to stair ascent**									
**- Collision of the foot during stair ascent**	1.00	1.00	1.00	1.00	1.00	1.00	1.00	1.00	VALIDATED
**General characteristics – items referring to stair descent**									
**- Difficulty in reaching the lower step during stair descent**	1.00	1.00	1.00	1.00	1.00	1.00	1.00	1.00	VALIDATED
**- Need for aids**	1.00	1.00	1.00	1.00	1.00	1.00	1.00	1.00	VALIDATED

* CVI: Content validity index

** Representativeness/relevance to the field of interest

## Discussion

Content validity is related to the robustness of score interpretations of an instrument and indicates the degree to which these scores measure what they claim to measure^35^ ^,^ ^36^. In the present study, three content validation phases were performed for the final set of items to obtain a consensus among the experts. According to Benson and Clark^29^, when absolute agreement is not reached for an item, the item must be revised until a consensus is reached. However, some items will never reach this standard despite several revisions and should therefore be excluded from the instrument. In this context, the first phase of content validation in the present study enabled a significant reduction in the size of the instrument with the exclusion of 41 items, including 15 that were excluded and 26 that were added to the Adopted Strategies Domain of the ascent and descent of stairs. Among the 15 items that were excluded, 6 belonged to the General Characteristics Domain and 9 belonged to the Adopted Strategies Domain. The items in the General Characteristics Domain that did not reach satisfactory levels of content validity were considered to have no value for the Instrument Content Domain. The 9 items excluded from the Adopted Strategies Domain assessed range of motion and compensations deemed incompatible with the qualitative kinematic analysis. In this first phase, important changes were also performed in the item wordings for better understanding, and therefore, the instrument was subjected to a re-evaluation of its content validity.

The results of the content validation process were determined by the CVI and Modified Kappa Coefficient. According to Polit et al.^33^, the items that presented CVI and Modified Kappa values above 0.70 were considered good and excellent. Thus, considering the final version of the instrument, it was observed that for the 38 items, 6 were validated in the first phase of content validity, 22 in the second phase and ten in the third and final phase. Nevertheless, in the first validation phase, 57.5% of the items reached acceptable levels (above 0.70) of content validity and Modified Kappa Coefficients for the consistency characteristic, 56.2% for representativeness, 50% for relevance and 32.5% for clarity. However, corrections were suggested by experts, thus justifying a re-evaluation. In the second validation phase, 81.6% of the items reached acceptable levels (above 0.70) for the characteristics of consistency and representativeness, 65.8% for relevance and 71% for clarity. The final 10 items subjected to the third evaluation reached acceptable levels and were considered validated in the aspects evaluated.

The final version of the clinical assessment instrument of the ascent and descent of stairs for individuals with hemiparesis had 38 items divided into 8 descriptive items: 16 items in the General Characteristics Domain for the ascent and descent of stairs and 14 items in the Adopted Strategies Domain for the ascent and descent of stairs (Appendix 1). Each item was assessed by an ordinal, categorical scale ranging from zero to two points that corresponded to the best and worst performance, respectively. The total score of the instrument was calculated separately for the ascent (0-70 points) and descent (0-74 points) of stairs. Because of the long instrument format, the major items of each domain are shown in Appendix 1, and the full instrument may be obtained by contacting the authors.

The 38 items that constituted the final version of the instrument presented adequate content validity for assessing the qualitative kinematic characteristics and strategies adopted in the ascent and descent of stairs for individuals with hemiparesis. However, further analytical studies of other psychometric properties, such as inter- and intra-examiner reliability, internal consistency and criterion and construct validity are necessary and will be performed in the future.

## Appendix 1 The clinical assessment instrument of stair ascent and descent in individuals with hemiparesis – major items.


